# Home-Based Exercise Therapy in the Management of Intermittent Claudication: A Systematic Review and Meta-Analysis

**DOI:** 10.7759/cureus.39206

**Published:** 2023-05-18

**Authors:** Alice Twomey, Zahid Khan

**Affiliations:** 1 Podiatry, Homerton University Hospital NHS Foundation Trust, London, GBR; 2 Acute Medicine, Mid and South Essex NHS Foundation Trust, Southend on Sea, GBR; 3 Cardiology, Barts Heart Centre, London, GBR; 4 Cardiology and General Medicine, Barking, Havering and Redbridge University Hospitals NHS Trust, London, GBR; 5 Cardiology, Royal Free Hospital, London, GBR

**Keywords:** grading of recommendations, standard deviations, critical limb ischaemia (cli), the national institute for health and care excellence (nice), atherosclerotic cardiovascular disease, systematic review and meta-analysis, peripheral arterial disease (pad), supervised exercise therapy (set), home based exercise therapy, intermittent claudication

## Abstract

The current literature strongly supports the use of supervised exercise therapy (SET) as the first-line treatment for symptomatic peripheral arterial disease (PAD) such as intermittent claudication (IC). However, this form of treatment remains underutilised in clinical practice. The home-based exercise therapy (HBET), in which patients must conduct themselves unsupervised is generally less effective than SET in terms of improving functional walking capacity. Nevertheless, it may be a useful alternative where SET is unavailable. The objective of this systematic review is to determine the effectiveness of HBET in reducing symptoms of IC in patients with PAD.

Studies eligible for inclusion in this systematic review were parallel-group randomised controlled trials (RCTs) published in the English language that compared the effect of HBET to a comparator arm (SET or no exercise/attention control) in adults with PAD and IC. Studies were eligible if outcome measures were available at baseline and at 12 weeks of follow-up or more. The electronic databases PubMed, Google Scholar, and the Cochrane Library were searched from the earliest records up to January 2021. The Cochrane Collaboration risk of bias tool for RCTs (RoB 2) was used to assess the risk of bias in individual studies, and the Grading of Recommendations Assessment, Development, and Evaluation (GRADE) classification system was used to appraise the quality of evidence for each outcome across all studies. The primary investigator independently collected, pooled, and analysed the data. The data was then entered into the ReviewManager 5 (RevMan 5) software, and a meta-analysis was performed by using a fixed or random effects model depending on the presence or absence of statistical heterogeneity.

The review author identified seven RCTs involving a total of 754 patients which were included in this study. Overall, the risk of bias in the included studies was moderate. Even though the results were variable, this analysis supported the ability of HBET to improve functional walking capacity and self-reported quality of life (QoL) to an extent.

This review shows that a home-based exercise intervention with regular professional support and encouragement is beneficial in improving functional walking capacity as well as some aspects of QoL in patients with PAD and IC when compared to no exercise. However, when HBET is compared to hospital-based supervised exercise intervention, SET yields greater benefits.

## Introduction and background

A peripheral arterial disease (PAD) is a manifestation of atherosclerosis that occurs in the peripheral arterial vessels resulting in compromised tissue perfusion of the lower (and sometimes upper) limb. An estimated 20% of the United Kingdom (UK) population between the ages of 55 and 75 years [1-3] are thought to have PAD, with a higher incidence amongst those who smoke, people with coronary artery disease, and individuals with diabetes [4-7]. As PAD is an atherosclerotic cardiovascular disease (CVD), typical risk factors include smoking, obesity, physical inactivity, dyslipidaemia, hypertension, and diabetes. The prevalence of PAD increases with age and is generally more common in men than women. There is also some evidence to suggest that ethnicity may play a role in increasing PAD risk [8]. Intermittent claudication (IC) is the most common symptom of PAD [9] causing pain in the calf muscles and less commonly in the thigh and buttock. IC is caused by ischaemia resulting from arterial stenosis or occlusion and is a predictable and repeatable symptom [6,9] induced by walking or exercise and relieved by rest.

The pain associated with IC is thought to occur when the oxygen requirement of the working muscle exceeds the insufficient blood supply due to the obstructed arterial pathway [10]. The intensity of pain ranges from mild to severe and can lead to increased functional impairment [11], mobility loss, and reduced quality of life (QoL) [12]. While symptoms in most patients with IC remain stable, 20% will develop increasingly severe symptoms and go on to develop critical limb ischaemia (CLI) with approximately 1-2% eventually requiring lower limb amputation, with a higher risk in those with diabetes (5%) [7]. Furthermore, in addition to limiting exercise capacity and promoting physical decline, PAD in the lower limb is an indicator of increased risk of myocardial infarction (MI) and stroke, even when asymptomatic and after adjusting for conventional CVD risk factors [10,13,14]. Approximately 10-15% of those with IC will die from cardiovascular causes within five years and a further 20% will go on to have a non-fatal cardiovascular event [7].

Differential diagnoses of IC include nerve root compression, spinal stenosis, osteoarthritis, and compartment syndrome, among others [15]. Therefore, without the presence of PAD, a diagnosis of IC cannot be made. PAD is usually identified through medical history and physical examination findings as well as diagnostic testing. Physical signs and symptoms of PAD include impaired walking ability, diminished foot and leg pulses, cold feet/toes, and non-healing foot or leg wounds. Ischaemic rest pain, pallor on the elevation of the legs or dependent rubor, and lower extremity necrosis are signs of CLI which occurs in the advanced stages of PAD [16]. Multiple diagnostic tests can be used to assess for PAD including the universally known and commonly utilised ankle brachial pressure index (ABPI) which includes the use of a handheld Doppler ultrasound auscultation. Variation exists between authors regarding the interpretation of ABPI readings; however, it is generally accepted that a normal ABPI is between approximately 0.8 and 1.3. A ratio of <0.5 is suggestive of severe PAD, between 0.5 and 0.8 is suggestive of moderate PAD, and >1.3 may be suggestive of arterial calcification [17]. Patients with an ankle-brachial index (ABI) of ≤0.90 are twice more likely to suffer from coronary events, cardiovascular mortality, and total mortality over 10 years time. Other diagnostic tests include toe pressure brachial index, arterial duplex ultrasound, contrast‑enhanced MRI, and CT angiography [7,18]. With regards to staging the severity of IC, the two most commonly used classification systems are the Fontaine classification in Europe and the Rutherford classification in the United States of America (USA) [19]. Over 20% of patients with IC present with an MI or stroke after five years with a mortality rate of 10-15% [19].

The primary management of IC is a conservative approach involving modification of cardiovascular risk factors, optimal medical management of high blood pressure, serum lipid levels and pain symptoms, and importantly, exercise therapy. Even though the exact mechanisms underpinning the benefits of exercise on IC symptoms are not entirely understood, it is known to inhibit disease progression by stimulating collateral vessel generation, improving maximum oxygen intake and endothelial cell function, reducing systemic inflammation, and increasing the oxidative capacity of skeletal muscle [20]. Furthermore, exercise therapy benefits the overall cardiovascular health of patients with IC which is essential as this patient group is prone to becoming less physically active because of leg pain, leading to a sedentary lifestyle which in turn promotes disease progression, increasing cardiovascular risk [21]. Thus, the benefits of exercise in general for those with IC are irrefutable.

Supervised exercise therapy (SET) is a structured exercise programme that takes place in a hospital or community healthcare setting and usually involves supervised walking (by a trained healthcare professional) on a treadmill [22] with optimal programmes recommending sessions of 30 to 60 minutes, three times per week for three to six months [7,13,23]. SET is considered the ‘gold-standard’ therapy for IC due to the abundance of evidence supporting its ability not only to promote functional improvement but to slow disease progression and improve the overall cardiovascular health as well as QoL of this patient group [20]. However, despite the evidence and multinational clinical guidelines endorsing the first-line use of SET in patients with IC, it remains a largely underutilised treatment modality [7,13,22]. The National Institute for Health and Care Excellence (NICE) (2012) guidelines indicate that a supervised exercise programme involving two hours per week of supervised exercise for a period of three months should be offered to all people with IC. However, a study by Harwood et al. (2017) reported that only 41% of vascular units in the UK had access to SET for patients with IC [7,20]. This further highlights the disparity between current clinical practice and national clinical guidance. The reasons for this discrepancy include lack of funding, shortage of facilities, and issues with patient participation [23,24].

Although research suggests HBET is less efficacious than SET, some studies have shown HBET to have a positive impact on functional walking capacity and QoL compared to simple ‘go-home-and-walk’ advice or no exercise [1,25]. Since many patients do not have access to, or are unable to partake, in SET, HBET may be a suitable alternative treatment option for patients with IC.

## Review

Methodology

In the absence of SET programme availability, the primary investigator of this review was prompted to investigate alternative, lower-cost treatment options for IC in patients with PAD in their local East London area of work. This was the rationale for conducting this systematic review. The aim of this study was to systematically review the evidence and provide an overview of the effects of HBET on improving functional walking capacity and QoL in those with IC in PAD.

This review was conducted according to the standards outlined in the Preferred Reporting Items for Systematic Reviews and Meta-Analyses (PRISMA) statement [26,27]. The study has been registered with PROSPERO under registration number CRD42023415135.

Population

Studies involving adults aged 18 years or older and of any gender with any severity of PAD with symptomatic IC were included. Studies that involved participants with CLI defined as rest pain or tissue loss were excluded.

Intervention

Studies that compared HBET (i.e., structured walking advice accompanied by active monitoring and support by medically trained personnel either face-to-face or by telephone) with SET, simple ‘go-home-and-walk’ advice, or no exercise control were included. Studies involving other forms of home-based exercise for the treatment of IC were not included.

Outcomes of Interest

Studies where the primary outcome (pain-free walking distance or time) and secondary outcome (improvement in QoL) measures were available at baseline and after at least 12 weeks of follow‐up were included.

The inclusion and exclusion criteria for eligible studies are displayed in Table 1 as per the PICO(S) framework [28].

**Table 1:** Inclusion and exclusion criteria for eligible studies HBET: home-based exercise therapy, SET: standard exercise therapy, CLI: critical limb ischaemia, IC: intermittent claudication, QoL: quality of life, RCTs: randomised controlled trials, PAD: peripheral arterial disease

Study characteristic	Inclusion criteria	Exclusion criteria
Population	Adult patients (aged 18 years and older) of any gender with any severity of PAD with symptomatic IC	Patients <18 years of age, patients with CLI; defined as rest pain or tissue loss
Intervention	Those treated with HBET (i.e., structured walking advice accompanied by active monitoring and support by medically trained personnel either face-to-face or by telephone)	Those treated with SET, simple ‘go-home-and-walk’ advice, or surgical revascularisation
Comparator	Those treated with SET, simple ‘go-home-and-walk’ advice, or no exercise control	Those with no active comparator
Outcomes of interest	Primary: Increase in functional capacity (pain-free walking distance or time) where outcome measures were available at baseline and after at least twelve weeks of follow‐up. Secondary: Improvement in QoL	No primary or secondary outcomes of interest are reported
Settings	In the patient's own home or chosen environment to conduct HBET	Outpatient/community healthcare setting
Study design	Parallel group RCTs, all sample sizes	Non-clinical studies (e.g., editorial, letter to the editor, case series), systematic reviews, meta-analysis, observational studies
Publications	English language studies	Studies published in languages other than English

The electronic databases PubMed, Google Scholar, and the Cochrane Library were searched from the earliest records up to January 2022. A comprehensive literature search for studies investigating the effects of HBET on IC symptoms in patients with PAD was performed using the following keywords: ‘intermittent claudication’ OR ‘claudication’ OR ‘peripheral arterial disease’ AND ‘home-based exercise’ OR ‘non-supervised exercise therapy’ OR ‘unsupervised exercise’ AND ‘randomised controlled trial’ OR ‘clinical trial’. We screened the titles and abstracts of each of the retrieved articles and selected studies for inclusion via full-text evaluation. No automation tools were used in the process. The primary and secondary outcomes of interest for which data were sought for each study are described and defined in Table 2.

**Table 2:** Definitions of primary and secondary outcome measures for individual studies SF-36: short-form 36 general health survey, WD: walking distance, ACD: absolute claudication distance, WIQ: walking impairment questionnaire, PWT: peak walking time, MWD: maximal walking distance, PWT: peak walking time, MWT: maximum walking time, 6-min WD: 6-minute walking distance

Study	Primary Outcome of Interest: Functional Walking Capacity	Secondary Outcome of interest: Self-Reported QoL
Gardner et al. (2011) [29]	Measurement used: PWT on an incremental treadmill test (measured in seconds). Definition: The walking time at which the ambulation cannot continue due to maximal leg pain.	Measurement used: WIQ and the Medical Outcomes Study SF-36.
McDermott et al. (2018) [30]	Description of measurement used: 6-minute walking distance (6-min WD) via walking up and down a 100 m hallway (measured in meters). Definition: The maximum walking distance completed after six minutes.	Measurement used: WIQ and the Medical Outcomes Study SF-36.
McDermott et al. (2013) [31]	Measurement used: 6-min WD via walking up and down a 100 m hallway (measured in meters). Definition: The maximum walking distance completed after six minutes.	Measurement used: WIQ.
Regensteiner et al. (1997) [32]	Measurement used: PWT on an incremental treadmill test (measured in minutes). Definition: The time taken to reach the maximal level of claudication pain that limited exercise.	Measurement used: WIQ.
Savage et al. (2001) [33]	Measurement used: ACD on an incremental treadmill test (measured in meters). Definition: The walking distance at which pain becomes so severe that the participant is forced to stop.	Measurement used: The Medical Outcomes Study SF-36.
Collins et al. (2011) [34]	Measurement used: MWD on an incremental treadmill test (measured in meters). Definition: The walking distance at which pain becomes so severe that the participant is forced to stop.	Measurement used: WIQ and the Medical Outcomes Study SF-36.
Patterson et al. (1997) [35]	Measurement used: MWT on an incremental treadmill test (measured in minutes). Definition: The maximum walking time to limit claudication.	Measurement used: The Medical Outcomes Study SF-36.

The risk of bias assessment in the included studies was performed using the Cochrane Collaborations risk of bias tool for RCTs (RoB 2) (Cochrane, London, England) [3] which includes five domains and categorises studies into ‘high risk’, ‘moderate risk’, or ‘low risk’. An example of a completed risk of bias assessment form showing full details of how the risk of bias was assessed can be found in Table 3. Continuous data was analysed by determining mean differences (MDs) and 95% confidence intervals (CIs). Dichotomous data was to be analysed by determining risk ratios and 95% CIs had there been any. All data was analysed by use of means and standard deviations (SDs) and the unit of analysis was the individual participant.

**Table 3:** Quality assessment of each included study according to Cochrane RoB 2

Study	Bias arising from the randomisation process	Bias due to deviations from intended interventions	Bias due to missing outcome data	Bias in the measurement of the outcome	Bias in the selection of the reported result	Overall risk of bias judgement
Gardner et al. (2011) [29]	Low	Low	Low	Low	Some concerns (unclear risk)	Moderate
McDermott et al. (2018) [30]	Low	Low	Low	Low	Low	Low
McDermott et al. (2013) [31]	Low	Low	Low	Low	Low	Low
Regensteiner et al. (1997) [32]	Some concerns (unclear risk)	Some concerns (unclear risk)	Some concerns (unclear risk)	Low	Some concerns (unclear risk)	Moderate
Savage et al. (2001) [33]	Some concerns (unclear risk)	Some concerns (unclear risk)	Some concerns (unclear risk)	Low	Some concerns (unclear risk)	Moderate
Collins et al. (2011) [34]	Some concerns (unclear risk)	Low	Low	Low	Some concerns (unclear risk)	Moderate
Patterson et al. (1997) [35]	Some concerns (unclear risk)	Low	Low	Low	Some concerns (unclear risk)	Moderate

As all included studies were RCTs, a statistical meta-analysis was performed where possible on results from all trials to produce forest plots including an overall effect estimate and associated 95% CIs. When data was not available from the trial paper, the study was excluded from the review. If data for at least one of the predefined outcomes of interest of the review was available, the study was included, and available data was examined in the meta‐analysis where possible. The data was analysed using the ReviewManager 5 (RevMan 5) software (Cochrane, London, England) [5], a computer programme recommended by the Cochrane Collaboration for preparing and maintaining systematic reviews. A meta-analysis was performed by using a fixed effects model unless significant heterogeneity was detected, in which case a random effects model was used in the analysis. SDs for group means were calculated using standard errors and CIs when SDs were not directly reported by the individual study authors. Statistical heterogeneity was assessed by use of the chi-squared test and the I2 statistic. Heterogeneity was deemed significant if the p-value of the chi-squared test was <0.01 or the I2 was greater than 50%.

Sensitivity Analysis

Comparisons included (1) HBET vs no exercise control and (2) HBET vs SET. Both HBET and SET interventions consisted of 12-week (three-month) interventions or 24-week (six-month) interventions, and in one study, the HBET programme was nine months in duration. Due to the differences in comparisons and programme duration between studies, data was separated and analysed using separate analyses depending on the comparison and the duration of intervention.

Risk of Bias Assessment

The Grading of Recommendations, Assessment, Development, and Evaluations (GRADE) classification system [4] was used to assess the overall certainty of the body of evidence for each outcome across all studies. An initial rating was assigned based on the study design and then revised (i.e., upgraded or downgraded) depending on the following criteria: risk of bias, imprecision, inconsistency, indirectness, and publication bias. A final quality rating was then given for each outcome based on this revision. Reporting bias was only assessed as per the RoB 2 tool only [3]. Of the included studies, five were deemed to be at moderate risk of bias due to (1) a lack of clarity around the randomisation process including sequence generation and allocation concealment; (2) a dearth of information regarding participant and investigators' awareness of participant’s assigned intervention during the trial; (3) inadequate evidence regarding deviations from intended interventions; (4) insufficient information relating to missing outcome data; and (5) a lack of evidence to allow for reporting bias to be ruled out definitively. The remaining two studies were deemed to be at low risk of bias across all five domains (Table 3).

Results

Study Selection

**Figure 1:**
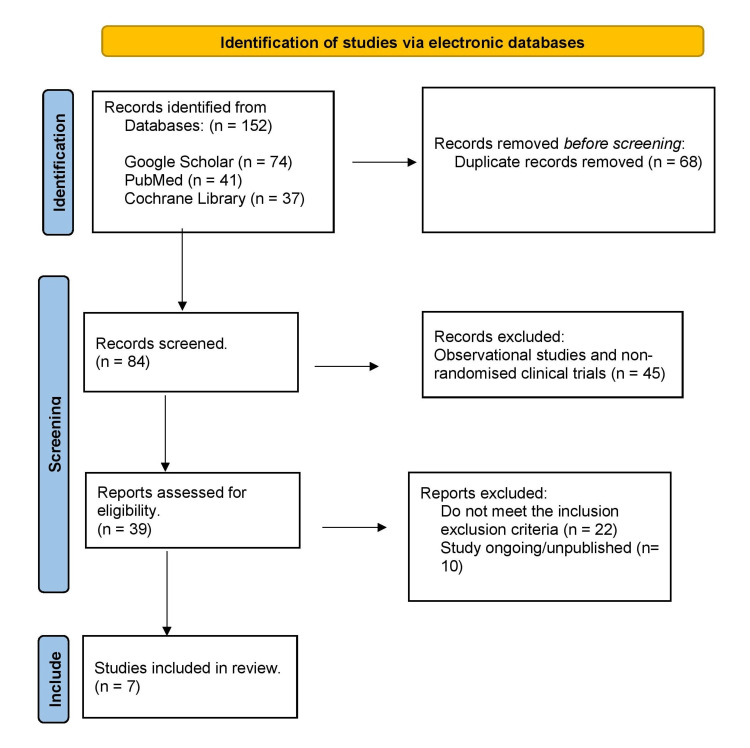
PRISMA flow diagram displaying the process of study selection for this systematic review PRISMA: Preferred Reporting Items for Systematic Reviews and Meta-Analyses [27]

The initial literature search yielded a total of 152 studies. After the removal of duplicates, 84 studies were screened, and 45 observational and non-randomised clinical trials were excluded. The remaining 39 studies were assessed for eligibility, of which 22 did not meet the inclusion criteria and 10 remain either ongoing or unpublished. Eventually, a total of seven RCTs that met the inclusion criteria were included in the review. Details of the search results can be found in Figure 1.

Study Characteristics

This review included the results of a total of 754 participants. The mean age of the participants ranged from 64 to 71 years, and the majority were male. The duration of the intervention and follow-up ranged from 12 weeks to nine months. The number of participants in the HBET groups varied from 10 to 99. The inclusion and exclusion criteria of the included studies varied greatly, though generally excluded those with severe comorbidities that would limit their ability to take part in HBET or would make HBET unfeasible.

In most of the included studies, researchers compared HBET with SET or attention/no exercise/usual care control apart from the trial by Gardner et al, 2011 [29] who conducted a three-arm parallel-group RCT comparing HBET with both SET and usual-care control. All studies outlined a reproducible method of HBET which predominantly consisted of walking three to five times per week at a self-selected pace until severe IC symptoms were experienced. They would then rest until symptoms subsided and continue this walking cycle until they had walked a total of approximately 45-50 minutes per session. In all studies, HBET was conducted in combination with varying levels of support for participants by a trained healthcare professional either face-to-face or via telephone. In these sessions, guidance and feedback were given to participants on their progress throughout the intervention period. In two of the trials, participants also used wearable activity monitors to monitor their physical activity [29,30].

While the majority of studies assessed their primary outcome of interest using a graded treadmill walking test, the two studies by McDermott et al. (2013) and McDermott et al. (2018) measured their primary outcome of interest by means of a six-minute walk distance test up and down a 100-ft hallway instead [30,31]. This method of measuring walking performance was reportedly chosen by the authors for several reasons including that (1) it is more closely correlated with the physical activity conducted during daily life in comparison to treadmill walking performance; (2) it is less commonly associated with balance and anxiety issues in older patients compared to treadmill walking; (3) it is not associated with a learning effect in the way treadmill walking is; and (4) it is well validated and is known to predict mobility loss and mortality in patients with PAD [31].

Results show considerable variation in outcome with some reporting walking distance and others reporting walking time. Authors reported various baseline haematological and biochemical measures for included participants as well as various cardiovascular risk factors and comorbid conditions. All studies reported ABPI measures at baseline with two studies also reporting a change in ABPI measures at follow-up [32,33]. All studies recorded self-reported QoL at baseline and follow-up; two studies used the walking impairment questionnaire (WIQ), two used the Medical Outcomes Study Short-Form 36 General Health Survey (SF-36) questionnaire, and three used a combination of both. Results of both the primary and secondary outcome measures were combined for meta-analysis in this systematic review where possible. A summary of the characteristics of included studies is displayed in Table 4.

**Table 4:** Summary of characteristics of included studies HBET: home-based exercise therapy, SET: supervised exercise therapy, PWT: peak walking time, WIQ: walking impairment questionnaire, SF: medical outcomes study short form, ACD: absolute claudication distance, MWT: maximum walking time, MWD: maximal walking distance: 6-min WD: 6-minute walking distance

Study	Study type	No. of participants randomised	No. of participants at follow-up	Age, years, mean ± SD	Male, %	Intervention	Control	Duration of intervention	Duration of follow-up	Outcome measures
Gardner et al. (2011) [29]	Parallel group RCT (three arm)	119	92	65 ± 11 HBET, 66± 12 SET, 65 ± 10 control	45 HBET, 45 SET, 54 control	HBET and SET	Usual care (no exercise)	12 weeks	12 weeks	Primary: PWT on an incremental treadmill test. Secondary: WIQ, SF-36
McDermott et al. (2018) [30]	Parallel group RCT	200	198	70.1 ± 10.6 HBET, 70.4 ± 10.1 control	46 HBET, 49 control	HBET	Usual care (no exercise)	9 months	9 months	Primary: 6-min WD. Secondary: WIQ, SF-36
McDermott et al. (2011) [31]	Parallel group RCT	194	178	69.3 ± 9.5 HBET, 71.0 ± 9.6 control	49 HBET, 48 control	HBET	Attention control	6 months	6 months	Primary: 6-min WD + MWT. Secondary: WIQ
Regensteiner et al. (1997) [32]	Parallel group RCT	20	20	64 ± 7	?	HBET	SET	12 weeks	12 weeks	Primary: PWT on an incremental treadmill test. Secondary: WIQ
Savage et al. (2001) [33]	Parallel group RCT	21	?21	66.3 ± 8.8	71	HBET	SET	6 months	12 weeks and 6 months	Primary: ACD on an incremental treadmill test. Secondary: SF-36
Collins et al. (2011) [34]	Parallel group RCT	145	126	66.5 ± 10.1	75	HBET	Attention control	6 months	6 months	Primary: MWD on an incremental treadmill test. Secondary: WIQ, SF-36
Patterson et al. (1997) [35]	Parallel group RCT	55	46 at 12 weeks, 38 at 6 months	69.3 ± 8.1	46.4 HBET, 59.3 SET	HBET	SET	12 weeks	12 weeks and 6 months	Primary: MWT on an incremental treadmill test. Secondary SF-36

Results of individual studies

HBET vs No Exercise Control

Four out of seven of the included studies compared HBET with no exercise control [29-31,34].

Peak Walking Time (PWT)/Maximum Walking Time (MWT)

Maximal Walking Distance (MWD)/Absolute Claudication Distance (ACD): The study by Collins et al. (2011) [34] was the only trial of those included in this comparison that reported on MWD. The authors noted that at six-month follow-up, there was no significant improvement noted in the MWD in the intervention group (P=0.60) or the control group.

6-Minute Walking Distance (6-Min WD): Of the four mentioned studies, two compared changes in 6-min WD between intervention and control groups [30,31]. Because the study authors reported their results at two different time points (six months and nine months respectively), it was not appropriate to combine these results for meta-analysis. McDermott et al. (2013) found that at six-month follow-up, there was a significant improvement noted in the 6-min WD in the intervention group (P <0.001) but not in the control group [32]. However, in the study by McDermott et al. (2018), no statistically significant difference in the mean change in 6-min WD from baseline to nine-month follow-up was found (P=0.31) [30].

**Figure 2:**
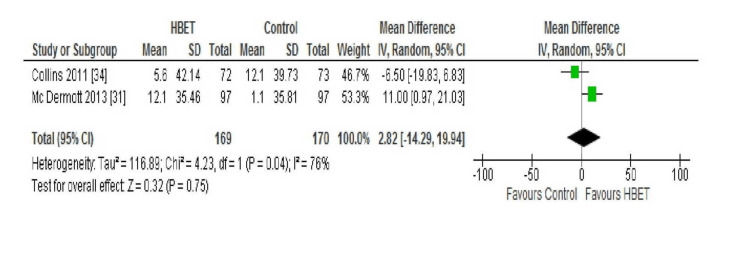
Forest plot displaying the combined results for WIQ distance score in the HBET intervention group vs the no exercise control group at six-month follow-up HBET: home-based exercise therapy, WIQ: walking impairment questionnaire

**Figure 3:**
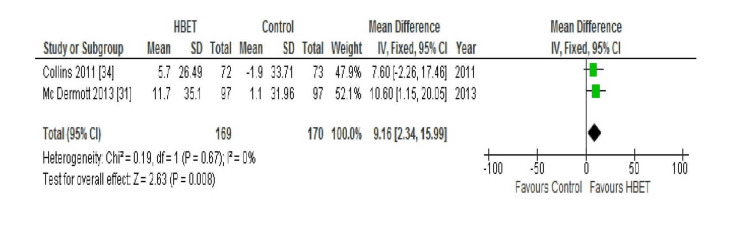
Forest plot displaying the combined results for WIQ speed score in the HBET intervention group vs the no exercise control group at six-month follow-up HBET: home-based exercise therapy, WIQ: walking impairment questionnaire

**Figure 4:**
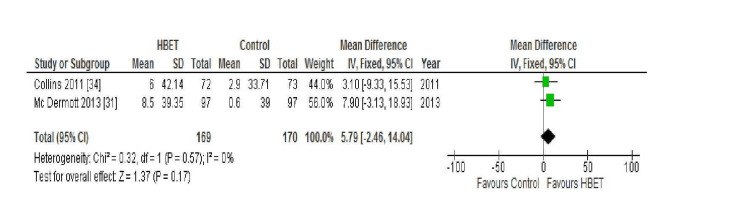
Forest plot displaying the combined results for WIQ stair climbing score in the HBET intervention group vs the no exercise control group at six-month follow-up HBET: home-based exercise therapy, WIQ: walking impairment questionnaire

QoL; WIQ: All four of the aforementioned studies compared the scores of the following three domains of the WIQ between baseline and follow-up: distance, speed, and stair climbing ability. McDermott et al. (2018) found no statistically significant difference in the mean WIQ distance (P=0.20), speed (P=0.69), or stair climbing (P=0.97) scores at nine months in either the intervention or the control group [30]. Conversely, Gardner et al. (2011) reported significant improvement in all three scores (distance P<0.05, speed P<0.05, and stair climbing P<0.05) at 12 weeks follow-up in the HBET intervention group (P<0.05) but not in the control group [29]. McDermott et al. (2013) and Collins et al. (2011) both reported their results at six-month follow-up for the three domains of this outcome which were, therefore, combined in a meta-analysis with the results displayed in Figures 2, 3, and 4 [31,34].

The WIQ distance score showed significant statistical heterogeneity (I2=76%) between studies; therefore, a random-effects model was used. McDermott et al. (2013) found a statistically significant improvement in the WIQ distance score (P<0.001) in the intervention group at six-month follow-up with no change in the control group [31]. However, no significant difference in the WIQ distance score at six-month follow-up was found by Collins et al. (2011) in either group [34]. Overall, the HBET group did not show a statistically significant improvement in the WIQ distance score between baseline and 6 months follow-up (MD 2.82 metres, 95% CI -14.29 to 19.94, P=0.75).

The WIQ speed score did not show significant statistical heterogeneity (I2=0%; Chi2=0.19) between studies; therefore, a fixed-effects model was used. Collins et al. (2011) and McDermott et al. (2013) [31] both found that at six-month follow-up, there was a significant improvement noted in the mean WIQ speed scores (P<0.05 and P=0.003, respectively) in the intervention group but not in the control group. Overall, the HBET group showed a statistically significant improvement in the WIQ speed score between baseline and six-month follow-up (MD 9.16 metres, 95% CI 2.34 to 15.00, P=0.008).

Again, for the outcome of the WIQ stair climbing score, a fixed effects model was used due to a lack of statistical heterogeneity between studies (I2=0%; Chi2=0.32). Neither study reported a significant improvement in participants' self-reported stair climbing ability in the intervention group from baseline to follow-up (McDermott et al. (2013) P=0.05; Collins et al. (2011) P=0.49) [31,34] or in the control group. Thus, overall, the HBET group did not show a statistically significant improvement in the WIQ stair climbing score either from baseline to six-month follow-up (MD 5.79 metres, 95% CI -2.46 to 14.04, P=0.17).

**Figure 5:**
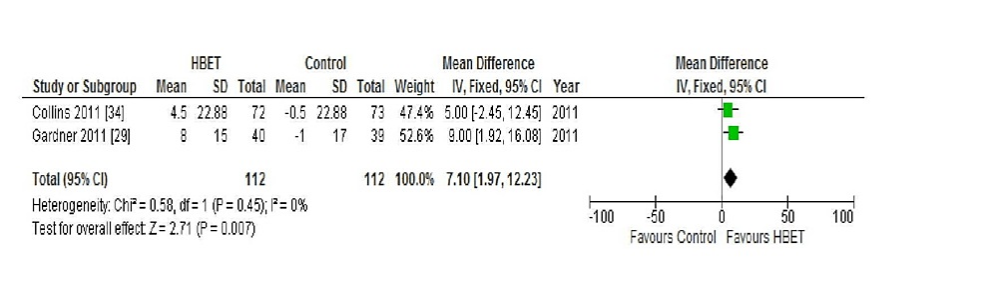
Forest plot displaying the combined results for the SF-36 physical function score in the HBET intervention group vs the no exercise control group at six-month follow-up HBET: home-based exercise therapy, SF-36: short-form 36 general health survey

QoL; SF-36 Questionnaire: Of the included studies, three reported on the SF-36 questionnaire which is made up of eight domains relating to self-reported QoL (physical function, role of physical function, bodily pain, general health, vitality score, social function, role of emotional health, and mental health). Gardner et al. (2011) and McDermott et al. (2018) only recorded results for one of the eight domains of this outcome (physical function score) and did so at 12 weeks and nine months, respectively [29,30]. Gardner et al. (2011) found a significant improvement in the SF-36 physical function score with HBET from baseline to follow-up (P<0.01) and no improvement in the control group [29]. Conversely, McDermott et al. (2018) did not find an improvement in the SF-36 physical function score at nine months in the HBET group (P=0.24) or control group [30]. Collins et al. (2011) at six-month follow-up reported on all eight domains of the SF-36 questionnaire and found that there was a significant improvement noted in the SF-36 mental health score only (P<0.05) in the intervention group [34]. No improvement was seen in any other of the seven domains in the intervention or the control group. It was only possible to combine the results of Gardner et al. (2011) and Collins et al. (2011) for the physical function score domain of this outcome for meta-analysis (Figure 5) [29,34].

For this outcome, a fixed effects model was used due to the lack of statistical heterogeneity detected between studies (I2 = 0%; Chi2 = 0.58). Overall, the HBET group did show a statistically significant improvement in the SF-36 physical function score from baseline to six-month follow-up (MD 7.10%, 95% CI 1.97 to 12.23, P = 0.007).

HBET vs SET

Four out of seven of the included studies compared HBET with SET [29,32,33,35].

PWT/MWT: Three out of the four studies reported on PWT/MWT [29,32,35] and all three did so at 12 weeks follow-up. Regensteiner et al. (1997) did not give numerical values for the MD or SDs for this outcome [32]. Nonetheless, the authors found that participants in the SET group improved their PWT by 137% (P<0.05) from baseline to follow-up, whereas no statistically significant improvement was found in the HBET group. Patterson et al. (1997) found that MWT improved in both SET and the HBET groups at the completion of a 12-week intervention (P<0.001), and this improvement was maintained at six months [35]. The authors did not give numerical values for the change score in MD or SDs for this outcome. Gardner et al. (2011) at 12-week follow-up also noted a significant improvement in PWT in both the HBET and the SET groups with no significant difference seen between groups [29]. It was not appropriate to combine the results of these three studies for meta-analysis due to the missing outcome data in two out of the three studies.

MWD/ACD: Savage et al. (2001) were the only authors that recorded results for ACD and did so at both 12-week and six-month follow-up [33]. At 12 weeks, an improvement was seen in ACD in both the HBET (P<0.009) and the SET (P<0.0001) groups, and although no further improvement was seen in ACD beyond 12 weeks, the improvement noted at 12 weeks was maintained at six months in both groups with no significant difference between groups.

QoL; WIQ: Two of the four studies that compared HBET with SET reported on this outcome and both did so at 12-week follow-up [29,32]. However, again, it was not possible to combine the results for meta-analysis due to the lack of appropriately reported results. Nevertheless, Regensteiner et al. (1997) found a significant improvement in WIQ distance (P<0.05) and speed (P<0.05) scores in the SET group but not in the HBET group [32]. The authors did not report on the WIQ stair climbing score. Conversely, Gardner et al. (2011) [29] noted that WIQ distance, speed, and stair climbing scores significantly improved in both intervention groups (distance: HBET P<0.05, SET P<0.05; speed: HBET P<0.05, SET P<0.01; stair climbing HBET P<0.05, SET P<0.001).

QoL; SF-36 Questionnaire: Three studies reported on the outcome of QoL using the SF-36 questionnaire [29,33,35]. All three recorded results at 12-week follow-up, while Savage et al. (2001) and Patterson et al. (1997) also did so at six months [33,35]. Gardner et al. (2011) [29] only reported on one of the eight domains of this outcome (physical function score) and found that the SF-36 physical function score improved significantly in both intervention groups (HBET P<0.01, SET P<0.01). The study by Patterson et al. (1997) concluded that there was a significant improvement in SF-36 physical function (P<0.01) and bodily pain (P<0.01) scores which was unchanged at six months in both groups [35]. All other domains showed no statistical significance from baseline to follow-up at 12 weeks or six months. As the authors did not report the exact figures in means and SDs, it was not possible to use these as part of a meta-analysis. Savage et al. (2001) found no change at 12 weeks or six months in any of the eight domains of the questionnaire in both groups [33].

Results of syntheses

Primary Outcomes

For the comparison of HBET vs control, results are largely variable between studies for the primary outcomes of interest. Of the two studies that assessed MWT/PWT at 12 weeks and six months follow-up, HBET did improve functional walking capacity. As for the outcome of MWD/ACD, only one study assessed this and did not find any improvement in functional walking capacity with HBET. Of the two studies that assessed 6-min WD, one found an improvement at six months with the use of HBET, and the other did not find an improvement with the use of HBET at nine months (summarised in Table 5).

**Table 5:** Summary of results of included studies comparing HBET with the control group for the primary outcomes of interest MWD: maximal walking distance, ACD: absolute claudication distance, PWT: peak walking time, MWT: maximum walking time

Comparison: HBET vs control			
Outcome	12-week follow-up	6-month follow-up	9-month follow-up
MWT/PWT	Gardner et al. (2011) [29]	McDermott et al. (2013) [31]	N/A
MWD/ACD	N/A	Collins et al. (2011) [34]	N/A
6-min WD	N/A	McDermott et al. (2013) [31]	McDermott et al. (2018) [30]
Note: J = statistically significant improvement seen in HBET intervention (P<0.05); L = no statistically significant improvement seen in HBET intervention (P>0.05); N/A = no study data for this outcome or time point			

For the comparison of HBET vs SET, again results vary significantly between studies. Of the three studies that assessed MWT/PWT at 12-week follow-up, HBET did improve functional walking capacity in two studies but not in the third. As for the outcome of MWD/ACD, only one study assessed this and found an improvement in functional walking capacity with HBET at 12 weeks which was maintained at six months. No study assessed 6-min WD for this comparison. Of all the studies that compared HBET with SET, those that saw an improvement with HBET for all outcomes also saw a similar, if not superior, improvement in functional walking capacity with SET (summarised in Table 6).

**Table 6:** Summary of results of included studies comparing HBET with SET for the primary outcomes of interest MWD: maximal walking distance, ACD: absolute claudication distance, PWT: peak walking time, MWT: maximum walking time

Comparison: HBET vs SET			
Outcome	12-week follow-up	6-month follow-up	9-month follow-up
MWT/PWT	Regensteiner et al. (1997) [32], Patterson et al. (1997) [35], Gardner et al. (2011) [29]	Patterson et al. (1997) [35]	N/A
MWD/ACD	Savage et al. (2001) [33]	Savage et al. (2001) [33]	N/A
6-min WD	N/A	N/A	N/A
Note: J = statistically significant improvement seen in HBET intervention (P<0.05); L = no statistically significant improvement seen in HBET intervention (P>0.05); N/A = no study data for this outcome or time point			

Secondary Outcomes

For the comparison of HBET vs control, results are significantly variable between studies and also for the secondary outcomes of interest. Of the four studies that recorded data on QoL using the WIQ for this comparison, all reported on all three domains (distance, speed, and stair climbing ability). One study found no improvement in any of the three domains with HBET intervention at nine months and another found improvement in all three domains with HBET at 12 weeks. Of the two that recorded data for this outcome at six months, one found an improvement in distance with HBET where the other did not; both found an improvement in speed; and neither noted an improvement in stair climbing ability. Of the three studies that recorded data on QoL using the SF-36 questionnaire, two recorded results for only one of the eight domains of this outcome: physical function. One found that HBET improved self-reported physical function and the other found that it did not. The one study that recorded results for all eight domains only found an improvement with HBET in self-reported mental health (summarised in Table 7).

**Table 7:** Summary of results of included studies comparing HBET with control for the secondary outcomes of interest SF-36: short-form 36 general health survey, WIQ: walking impairment questionnaire

Comparison: HBET vs control			
Outcome	12-week follow-up	6-month follow-up	9-month follow-up
WIQ distance	Gardner et al. (2011) [29]	McDermott et al. (2013) [31], Collins et al. (2011) [34]	McDermott et al. (2018) [30]
WIQ speed	Gardner et al. (2011) [29]	McDermott et al. (2013) [31], Collins et al. (2011) [34]	McDermott et al. (2018) [30]
WIQ stair climbing ability	Gardner et al. (2011) [29]	McDermott et al. (2013) [31], Collins et al. (2011) [34]	McDermott et al. (2018) [30]
SF-36 physical function	Gardner et al. (2011) [29]	Collins et al. (2011) [34]	McDermott et al. (2018) [30]
SF-36 role of physical function	N/A	Collins et al. (2011) [34]	N/A
SF-36 bodily pain	N/A	Collins et al. (2011) [34]	N/A
SF-36 general health	N/A	Collins et al. (2011) [34]	N/A
SF-36 vitality score	N/A	Collins et al. (2011) [34]	N/A
SF-36 social function	N/A	Collins et al. (2011) [34]	N/A
SF-36 role of emotional health	N/A	Collins et al. (2011) [34]	N/A
SF-36 mental health	N/A	Collins et al. (2011) [34]	N/A
Note: J = statistically significant improvement seen in HBET intervention (P<0.05); L = no statistically significant improvement seen in HBET intervention (P>0.05); N/A = no study data for this outcome or time point			

For the comparison of HBET vs SET again, results are significantly varied between studies. Two studies recorded data on QoL using the WIQ for this comparison. One only reported on two domains (distance and speed) and found no significant improvement in either with HBET. The other noted improvements in all three domains (distance, speed, and stair climbing ability) with HBET. Of the three studies that reported on QoL using the SF-36 questionnaire, two found an improvement in self-reported physical function, one found an improvement in bodily pain, and one found no improvement in any domain with HBET (summarised in Table 8).

**Table 8:** Summary of results of included studies comparing HBET with SET for the secondary outcomes of interest WIQ: walking impairment questionnaire, SF-36: short-form 36 general health survey

Comparison: HBET vs SET			
Outcome	12-week follow-up	6-month follow-up	9-month follow-up
WIQ distance	Gardner et al. (2011) [29], Regensteiner et al. (1997) [32]	N/A	N/A
WIQ speed	Gardner et al. (2011) [29], Regensteiner et al. (1997) [32]	N/A	N/A
WIQ stair climbing ability	Gardner et al. (2011) [29]	N/A	N/A
SF-36 physical function	Gardner et al. (2011) [29], Savage et al. (2001) [33], Patterson et al. (1997) [35]	Savage et al. (2001) [29], Patterson et al. (1997) [35]	N/A
SF-36 role of physical function	Savage et al. (2001) [33], Patterson et al. (1997) [35]	Savage et al. (2001) [29], Patterson et al. (1997) [35]	N/A
SF-36 bodily pain	Savage et al. (2001) [33], Patterson et al. (1997) [35]	Savage et al. (2001) [29], Patterson et al. (1997) [35]	N/A
SF-36 general health	Savage et al. (2001) [33], Patterson et al. (1997) [35]	Savage et al. (2001) [29], Patterson et al. (1997) [35]	N/A
SF-36 vitality score	Savage et al. (2001) [33], Patterson et al. (1997) [35]	Savage et al. (2001) [29], Patterson et al. (1997) [35]	N/A
SF-36 social function	Savage et al. (2001) [33], Patterson et al. (1997) [35]	Savage et al. (2001) [29], Patterson et al. (1997) [35]	N/A
SF-36 role of emotional health	Savage et al. (2001) [33], Patterson et al. (1997) [35]	Savage et al. (2001) [29], Patterson et al. (1997) [35]	N/A
SF-36 mental health	Savage et al. (2001) [33], Patterson et al. (1997) [35]	Savage et al. (2001) [29], Patterson et al. (1997) [35]	N/A
Note: J = statistically significant improvement seen in HBET intervention (P<0.05); L = no statistically significant improvement seen in HBET intervention (P>0.05); N/A = no study data for this outcome or time point			

Certainty of evidence

Due to the low number of RCTs included in this review and the inability to meta-analyse and produce an overall effect estimate for many of the outcomes, it was not possible to apply the GRADE approach [4] to each outcome assessed, as was intended. However, an assessment of certainty was made for each outcome based on the risk of bias, inconsistency, indirectness, imprecision, and publication bias as displayed in Table 9. All outcomes started out as having a high level of certainty as all included studies were RCTs.

**Table 9:** Outcome quality assessment profiles HBET: home-based exercise therapy, SF-36: short-form 36 general health survey, PWT: peak walking time, WIQ: walking impairment questionnaire, ACD: absolute claudication distance, MWT: maximum walking time, MWD: maximal walking distance, 6-min WD: 6-minute walking distance, GRADE: Grading of Recommendations Assessment, Development, and Evaluation

Outcome	Quality/certainty of evidence (GRADE)
PWT/MWT	⊕⊕ Low
ACD/MWD	⊕⊕ Low
6-min WD	⊕⊕⊕⊕ High
QoL: WIQ	⊕⊕⊕ Moderate
QoL: SF-36	⊕⊕⊕ Moderate

Discussion

Based on moderate quality evidence, this systematic review demonstrated that a home-based exercise intervention, with regular professional support and encouragement, is beneficial for improving functional walking capacity as well as some aspects of QoL in patients with IC and PAD when compared to placebo or usual care (i.e., no exercise). However, when comparing HBET to SET, the latter was demonstrated to be the more superior therapy as walking ability improved even further with a structured hospital-based supervised exercise intervention. Overall, the data presented generally confirms the findings of previous systematic reviews conducted on the same topic.

This review found that walking distances and walking times generally improved with HBET and with SET when assessed at 12 weeks, which were sustained at 6 months. As for QoL, the results were varied. It was found that HBET improved self-reported walking speed (based on the WIQ) at six-month follow-up and self-reported physical function (based on the SF-36 physical function score) at six-month follow-up. However, it remains unclear from this review whether or not HBET improves any other aspect of self-reported QoL in patients with PAD and IC at any time point. It is also uncertain from this review whether improvements would be sustained beyond six months with HBET or SET which raises questions with regard to the longer-term benefits of exercise therapy in the treatment of IC in patients with PAD.

It is well evidenced that structured hospital-based SET on a treadmill improves walking performance and QoL in patients with PAD and IC, in addition to improving cardiovascular health and reducing morbidity and mortality. That said the barriers hindering patient participation in SET are multidimensional. For example, there are not many PAD-specific supervised exercise programmes in existence, and of those that do exist, they are based in larger vascular hubs with comparatively poor provisions for patients who do not live locally [36,37]. Therefore, associated travel and accessibility constraints are partly responsible for poor patient participation in SET. Additionally, compliance is known to be poor amongst patients with IC. Patients with PAD are known to have reduced physical activity levels compared to their healthy age-matched counterparts, and it is quite possible that these adherence issues are linked to the poor lifestyle choices and behaviours that led to the development of debilitating IC in the first place [38,39]. It is important to also note that despite the clinical trial evidence and clinical guidance in support of the provision of SET, hospital commissioning groups in the National Health Service in the UK have not prioritised funding hospital-based supervised exercise programmes as a primary treatment for IC prior to EVR. This is particularly interesting given the significant cost savings associated with SET compared to the latter [40]. It has been suggested that reasons for this may include uncertainties around the benefit of SET vs unsupervised exercise; lack of clarity regarding the optimum form of SET; and a lack of large, adequately powered randomised trials [39]. However, it is argued that while further larger RCTs would indeed be helpful, insufficient data is not the issue here but a lack of incentive for hospitals to prioritise SET prior to surgical intervention [39,41].

With regards to HBET, there remains a lack of high-quality clinical trial data on the effectiveness of this form of exercise therapy in improving walking ability and reducing pain symptoms in IC. It is, however, as this review suggests, certainly a better alternative than simple 'go-home-and-walk' advice, and results of more recent and ongoing trials are promising [42-44].

It is worth considering that many individuals with IC are elderly with other comorbid conditions which may render walking an unfeasible mode of exercise to conduct despite being motivated to take part in exercise therapy. A growing body of research has demonstrated that alternative modes of aerobic exercise may be equally as beneficial as walking for reducing IC symptoms such as cycling, strength training, and upper body ergometry [45,46]. Safety is another aspect of exercise therapy for this patient group that must be considered as patients with IC are at increased risk of cardiovascular events.

A key difference between McDermott et al. 2013 and 2018 studies was the difference in improvement in 6-min WD in 2013. The McDermott et al. 2018 study demonstrated no significant improvement in 6-min WD in the HBET intervention group compared to the control group at nine-month follow-up. The mean change in the 6-min WD was 5.5 m in the intervention group vs 14.4 m in the control group at nine-month follow-up. The participants in 2013 had a significant increase in 6-min WD in the intervention group vs the control group with a MD of 53.5 m and a maximum treadmill walking time MD of 1.01 minutes. With regards to improving the motivation of patients to engage with treatment, McDermott and Polonsky (2016) highlighted that modern technology, such as tele-coaching or tele-monitoring via wearable monitors, could assist patients in successfully conducting HBET [47]. They explained that the increasing availability of low-cost activity monitors, as well as the use of electronic health records and online patient portals, makes HBET a more feasible treatment option. This in time will potentially allow HBET to become more accessible to this patient group, although further clinical trial data as well as the creation and standardisation of clinical guidelines outlining best clinical practice for HBET would be a necessity in the first instance.

Finally, the abovementioned studies are mostly from developed countries. Although the prevalence of lower limb PAD is higher in high-income countries compared to low- and middle-income countries (7.4% vs 5.1%), if we take the population size into consideration, then most individuals with PAD (72.9%) were in low- and middle-income countries [48,49]. Additionally, the prevalence of PAD has increased by 18% in high-income and 58% in low-income countries which account for a 45% global increase from 2000 to 2015 [48].

Study limitations

A major limitation of the study is the small number of studies included in the review, significant variability between studies, and the lack of appropriate outcome data. It was not possible to conduct a meta-analysis for most outcomes. Furthermore, significant variations in pooled outcomes at all time points lead to the need for data analysis to be conducted at specific time points in an attempt to address heterogeneity (e.g., at 12 weeks and at six-month post-intervention). The bias present within the individual-included studies was frequently challenging to assess. Secondly, expanding the inclusion criteria to also include observational and non-clinical trials, for example, may have also allowed this study to be more comprehensive. However, the inclusion of only seven eligible studies out of 152 identified in the initial search of three major search engines highlights the overall scarcity of available literature on this topic. There is a slight variation in the classification of PAD between the NICE and the European Society of Cardiology (ESC) guidelines. According to the ESC guidelines, an ABI <0.90 is indicative of the presence of PAD, ABI between 0.90 and 1.00 is borderline, ABI between 1.00 and 1.40 is normal, and ABI >1.40 is abnormally high, whereas, in the NICE guidelines, an ABPI ratio of <0.5 suggests severe PAD. An ABPI ratio of >0.5 and <0.8 suggests the presence of arterial disease or mixed arterial/venous disease, and values between 0.8 and 1.3 are considered to be normal, and values of >1.3 are suggestive of the presence of arterial calcification in patients with diabetes, rheumatoid arthritis, systemic vasculitis, and chronic renal failure [13,50]. This classification may have affected the outcome of some studies.

Implications for future research

This review highlighted significant variations in several of the characteristics of the included studies. As previously reported, the risk of bias between studies was varied, as was the way in which studies reported their outcome data. In view of the latter, it is suggested that future research focuses on larger, more robust clinical trials with improved methodological transparency and standardisation of HBET intervention protocols. As previously mentioned, this would allow for the provision of standardised clinical guidelines outlining the best evidence-based practice for HBET in patients with IC. It would also be beneficial for future studies to assess the longer-term benefits of HBET (>6 months) in this patient group.

## Conclusions

This review demonstrated with moderate quality evidence that hospital-based supervised walking exercise therapy remains the ideal treatment option for IC in patients with PAD, but if unavailable, a well-structured home-based exercise intervention should be considered given its ability to improve functional walking capacity and some aspects of QoL. This was demonstrated in the studies included in this systematic review in both HBET vs control and HBET vs SET. This inexpensive and more convenient treatment option may be the way forward in treating IC in those who are suitable candidates for exercise therapy if facilitated effectively. Further larger size randomised controlled trials would be useful to assess the effectiveness of home-based exercise intervention which could be a feasible long-term option for patients.
